# Coming of Age for the Photoreceptor Synapse

**DOI:** 10.1167/iovs.62.12.24

**Published:** 2021-09-22

**Authors:** Ellen Townes-Anderson, Eva Halasz, Weiwei Wang, Marco Zarbin

**Affiliations:** 1 Department of Pharmacology, Physiology, and Neuroscience, Rutgers New Jersey Medical School, Newark, New Jersey, United States; 2 Schepens Eye Research Institute and Massachusetts Eye and Ear Infirmary, Department of Ophthalmology, Harvard University, Boston, Massachusetts, United States; 3 Institute of Ophthalmology and Visual Science, Rutgers New Jersey Medical School, Newark, New Jersey, United States

**Keywords:** photoreceptor morphology, plasticity, RhoA-ROCK, synapse, retinal detachment

## Abstract

**Purpose:**

To discuss the potential contribution of rod and cone synapses to the loss of visual function in retinal injury and disease.

**Methods:**

The published literature and the authors’ own work were reviewed.

**Results:**

Retinal detachment is used as a case study of rod spherule and cone pedicle plasticity after injury. Both rod and cone photoreceptors terminals are damaged after detachment although the structural changes observed are only partially overlapping. For second-order neurons, only those associated with rod spherules respond consistently to injury by remodeling. Examination of signaling pathways involved in plasticity of conventional synapses and in neural development has been and may continue to be productive in discovering novel therapeutic targets. Rho kinase (ROCK) inhibition is an example of therapy that may reduce synaptic damage by preserving normal synaptic structure of rod and cone cells.

**Conclusions:**

We hypothesize that synaptic damage contributes to poor visual restoration after otherwise successful anatomical repair of retinal detachment. A similar situation may exist for patients with degenerative retinal disease. Thus, synaptic structure and function should be routinely studied, as this information may disclose therapeutic strategies to mitigate visual loss.

Sensory receptors, and photoreceptors in particular, are exquisitely complex cells. At one end, a photosensitive organelle, the outer segment, which transduces energy from visible light into a membrane potential change, connected by a modified cilium, which helps create the membranous outer segment, to an inner segment where metabolic needs are met and proteins synthesized, then the cell body with the nucleus, and a fiber that is both axon- and dendrite-like extends to the final compartment, a presynaptic terminal. But not a conventional terminal; it is a ribbon synapse highly specialized to deliver glutamate in ever changing amounts, in response to light levels, to multiple postsynaptic cells. However, when describing the effects of disease or injury on this complex receptor, reports most often focus on the changes in the outer segment: are the membranous disks disorganized, how many are gone, and has the length of the outer segment returned to normal? We would like instead to turn the spotlight to the synaptic terminal, the first synapse in the visual pathway without which no sensation of light would occur.

## Rod Spherules

More than 30 years ago, in a cat model of retinal detachment, changes in the first synapse were noted in response to the detachment injury.^1^^,^^2^ Because of the ease of immunocytochemical detection, more is known about rod synapses after detachment: in contrast to cone terminals, rod presynaptic terminals retain their characteristic proteins and synaptic markers while undergoing dramatic movements in response to injury, uncoupling from their postsynaptic partners and withdrawing into the outer nuclear layer (ONL).^3^ After retraction of the spherule, the rod cell's postsynaptic partners react; rod bipolar dendrites sprout, extending into the outer nuclear layer, and horizontal cell axons grow extensively in the outer and inner retina.^2^^,^^5^ Surprisingly, and in contrast to the regeneration of outer segments, reattachment of the retina does not restore the outer plexiform layer. In fact, rod terminals continue to exist in the outer nuclear layer weeks after reattachment.^5^ In addition, new structural plasticities occur. At rod terminals, neuritic sprouts, visible because of the abnormal diffusion of opsin throughout the rod cell plasma membrane, extend into the inner nuclear layer and develop presynaptic varicosities. Although some normal synaptic structures, like ribbons, have been described in the varicosities along the rod sprouts, normal synaptic contacts with other retinal neurons do not form.^5^

**Figure 1.**
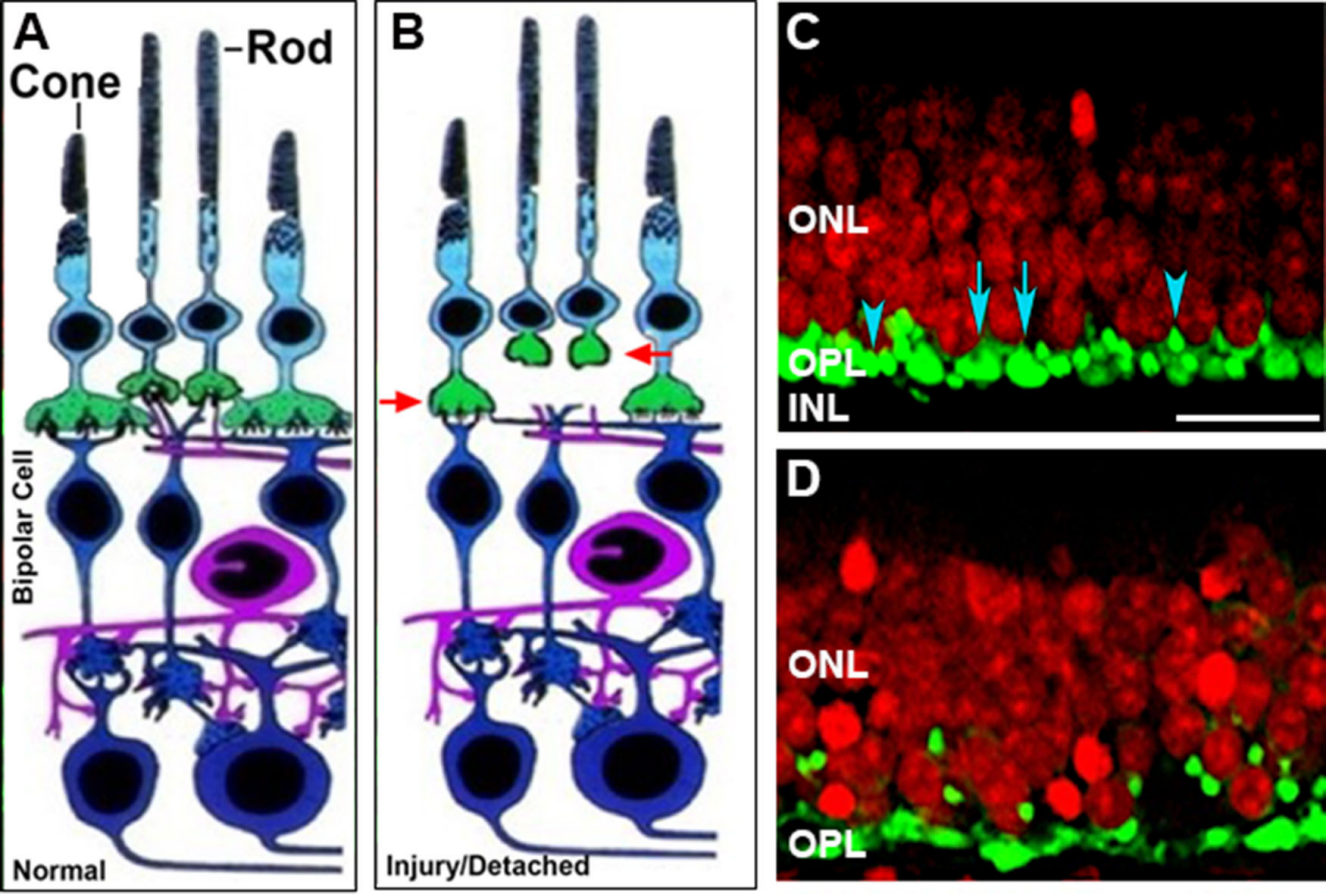
Injury-induced synaptic disjunction. **A.** Diagram of normal retina, modified from Dowling and Boycott 1966.^86^ **B**. After detachment, rod axons and terminals retract from the outer plexiform layer and cone terminals round up (*red arrows*). **C**. Detached retina labeled for synaptic proteins (SV2, *green*) and nuclei (*red*). *Top*, within hours after detachment rod (*blue arrowheads*) and cone (*blue arrows*) become rounded in shape. **D**. 24 hours later retracted rod spherules are present in the outer nuclear layer while pedicles appear flattened. *Scale bar*, 10 µm. **C-D**. Porcine retina maintained in vitro, modified from Fontainhas and Townes-Anderson 2011.^40^

In our more recent studies on retinal detachment using a pig model, we also observed many of these synaptic changes (Fig. 1). Our work has looked at shorter timeframes and therefore has added new information: retraction of the rod presynaptic terminal occurs within hours of detachment, in other words, very quickly,^6^ and rod synaptic reactions occur in many places throughout the retina including more than a centimeter away from the detachment in areas that remain attached.^6^^–^^8^ It appears that there is a wave of change across most of the retina in response to the local injury. Two to seven days later, when the retina has spontaneously reattached, rod terminals remain in the outer nuclear layer, although in reduced numbers compared to two hours after detachment^8^ (unpublished data, 2020). Bipolar cell sprouting in our model begins about two days after detachment/reattachment.^8^

Both the previous retinal detachment studies and our own suggest that continued disruption of synapses contribute to the visual disturbances, including lower acuity, consistently observed after anatomically successful reattachment surgery.^9^^–^^16^ Indeed, we saw a high correlation between the amount of rod synaptic retraction, determined by misplaced synaptic vesicle labeling, and the reduction in scotopic responses two days after detachment/reattachment.^8^ In other words, in addition to damaged outer and inner segments, loss of synapses due to synaptic remodeling can contribute to the lack of physiological recovery after retinal detachment.

### Genetic Retinal Degeneration

Are rod synaptic changes unique to retinal detachment injury? Published descriptions of synaptic injury in retinal degeneration are now quite common. Retracted rod presynaptic terminals are found in the outer nuclear layer in human retinitis pigmentosa (RP),^17^ in models of congenital stationary blindness,^18^ glaucoma,^19^ retinal degeneration (autosomal recessive RP,^20^ X-linked RP^21^^,^^22^), oxygen-induced retinopathy (OIR),^23^ retinoschisis,^24^ and in human and animal models of normal aging and age-related macular degeneration (AMD).^25^^–^^28^ Bipolar and horizontal cell sprouting has been described in human RP,^17^ AMD and aging,^25^^,^^26^ and models of RP,^21^^,^^22^ congenital stationary night blindness,^18^ and AMD.^27^^,^^28^ Finally, rod neuritic sprouts in the inner retina have been found in multiple subtypes of human RP,^17^^,^^29^^,^^30^ in animal models of RP,^31^^–^^33^ in AMD,^34^ in rod/cone dysplasia,^35^ and after laser damage.^36^ Thus, we should add rod synaptic change and loss to the set of problems to be considered and addressed in new therapies for retinal disease.

### Sequence of Synaptic Change

If one examines the list of observations for rod terminal retraction, sprouting by bipolar and horizontal postsynaptic partners, and rod neuritic sprouting, it is evident that these phenomena frequently occur in the same injury or disease, suggesting that the neurons involved in the first synapse of the visual system work as a functional unit not only in normal physiology but also in pathology with a stereotypical response. We have reported that rod terminal retraction occurs first^6^ in response to detachment. In retinal disease some have suggested that the entire synaptic complex is retracted into the ONL.^18^ However, examination of the very early events, which might show that retraction of the spherule occurs first, is often absent. Alternatively, the nature or the magnitude of the perturbation in the circuitry could induce different reactions. Sprouting of postsynaptic cells may be sequential. In a mouse retina, mutant for the presynaptic scaffolding protein bassoon, horizontal cell sprouting occurs before rod bipolar neuritic growth.^37^ Finally, it seems that rod neuritic sprouting into the inner retina occurs after sprouting of the secondary neurons as it is a phenomenon seen after retinal reattachment, long after detachment-associated changes have occurred. In the mouse, rod cell sprouting does not occur, perhaps because of rapid rod cell death in most mouse retinal degenerations.^29^ With this scenario in mind, it is tantalizing to think that if rod terminal retraction is blocked, no further remodeling and synaptic disruption would occur in the rod pathway.

## Cone Pedicles

In human cone cell disease, not all functional visual loss correlates with degenerative outer/inner segment changes: in human X-linked RP (XLRP) with mutations in the RP-GTPase regulator gene (*RPGR*), loss of retinal sensitivity to 543 nm light compared with cone inner segment thickness and cell density reductions as seen with high resolution microperimetry, was greater than expected.^38^ Recently, in the rd9 mouse, a model for XLRP, rod cell spherule retraction and postsynaptic cell sprouting were described, and, despite the normalcy of cone cell morphology, reduction in photopic b-wave responses was reported.^21^ Similarly, in two canine models of RP with *RPGR* mutations, substantial rod circuitry remodeling was reported, which caused reduced retinal function, although no cone synaptic changes were observed.^22^ Again, studies of retinal detachment may lead the way to an enhanced understanding of photoreceptor degeneration.

More than a decade ago, changes in cone cell synapses after detachment were described in a feline model of retinal detachment. They included rounding or flattening of the cone pedicles, loss of synaptic invaginations, and reduction in number and size of ribbons.^5^ In our pig model, reduction of ribbon length and loss of invaginations occur within hours after detachment along with shape changes to the pedicles (Fig. 1).^39^^,^^40^ It should be noted that rod terminals also exhibit shallow invaginations and some reduction in ribbon size after detachment, but these changes are less dramatic than the retraction of the spherule resulting in synaptic disjunction. In contrast, cone synapses show no patent synaptic disjunction. However, the cone axons can appear tortuous, perhaps due to movement of the cone cell body inwards into the outer plexiform layer.^5^ Changes at the molecular level accompany the pedicles’ morphological changes. In contrast to rod cells, most molecular markers specific to cone cells disappear after three to seven days of detachment (i.e., cone opsins, calbindin D, GCAP-1).^4^ Although cone opsin mRNA expression returns after reattachment,^41^ the structural integrity of cone synapses after reattachment is unknown. If rod synapses are a guide, it is likely that some changes in cone synapses remain after reattachment. Consistent with this hypothesis, patients with retinal detachments present with reduced photopic b-wave responses months after anatomically successful reattachments.^42^^–^^45^ More work is needed to understand cone synaptic plasticity during detachment and disease and the role of rod and cone synaptic changes among patients with persistent visual loss after outer and inner segment regeneration, whether arising from RP-like disease, retinal detachment, or blunt trauma.

## Mechanisms of Synaptic Plasticity

What might be the mechanisms and therefore possible therapeutic targets for control of photoreceptor synaptic plasticity after injury and during disease? We speculated that much could be learned from previous work on the plasticity of conventional synapses during learning and memory, where signaling pathways are well known.^46^ Glutamate, calcium, and the cyclic nucleotides, cAMP and cGMP, are among the main actors. Since photoreceptors have no glutamate NMDA receptors, we assessed calcium and cyclic nucleotides. Calcium plays a role in detachment-induced rod synaptic retraction in vitro and blocking L-type channels reduced rod cell plasticity of isolated rod cells^47^^,^^48^ and intact neural retina in culture.^40^ Cyclic AMP via phosphorylation of the transcription factor cAMP response-element binding protein (CREB, another player in activity-dependent synaptic plasticity^46^) also prevents retraction and can stimulate rod sprouting in intact neural retina in vitro.^49^^,^^50^ We have suggested that activation of rod opsin that diffuses along the inner segment cell membrane in injury and disease, known as mislocalized opsin, is able to stimulate adenylyl cyclase to increase cAMP and CREB activity.^50^^,^^51^ For cone cells, blocking their cGMP-gated calcium channels prevented the formation of presynaptic varicosities in isolated cone cells whereas addition of the channel agonist 8-bromo-cGMP increased varicosity formation.^48^ Although there is currently no evidence of new cone synapse formation after detachment or reattachment, remodeling, including development of a small number of synaptic structures, has been observed in mouse cones after partial loss of cone cells by diphtheria toxin.^52^ Furthermore, activation of soluble guanylyl cyclase, to increase cGMP, stimulated neuritic sprouting of isolated cone cells^53^ suggesting an explanation for the unusual cone cell sprouting observed in an autosomal recessive form of RP characterized by high cGMP levels in the outer retina.^54^^,^^55^

**Figure 2.**
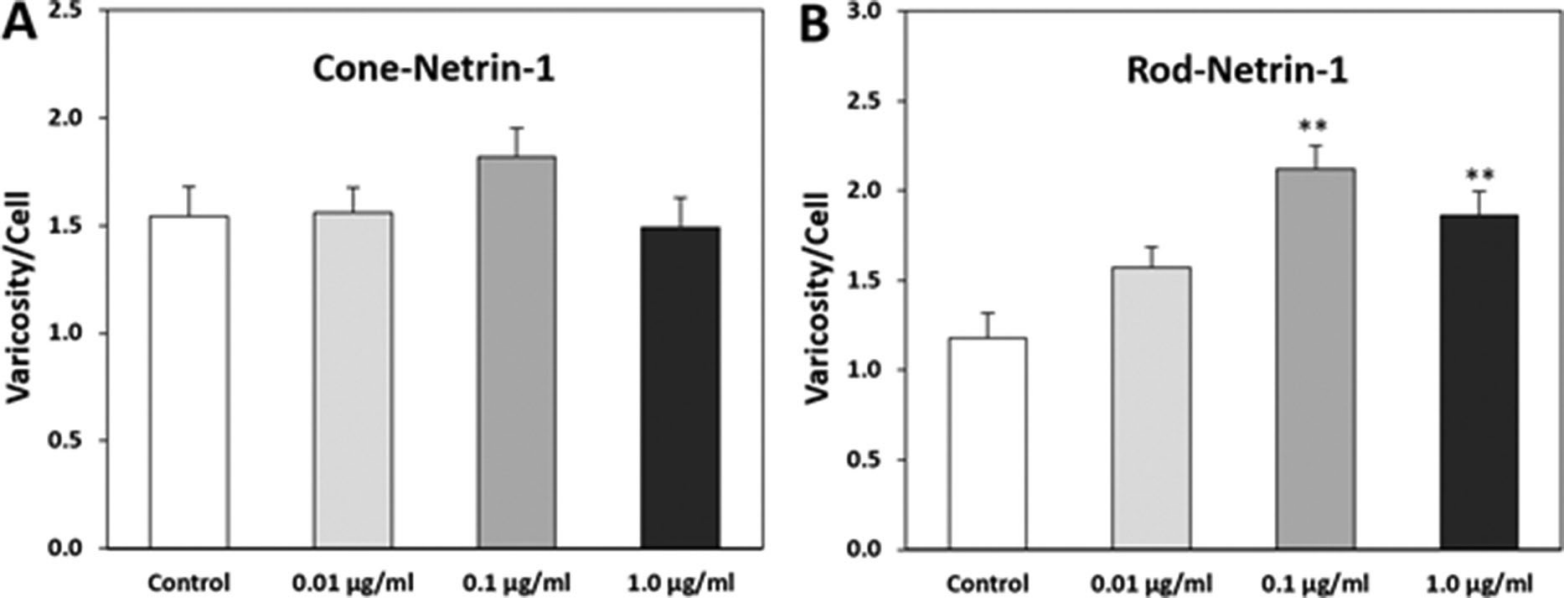
Netrin increases the formation of presynaptic varicosities in isolated rod cells. Data from adult salamander retinal cell cultures. Netrin was added to the culture medium at indicated concentrations. After three days in culture, the higher doses of netrin-1 significantly increase the production of varicosities by rod (**B**) but not cone (**A**) cells. Cultures were stained for rod opsin and synaptophysin to highlight presynaptic formation. **P < 0.001, + SEM, *n* = 800 cells, 16 cultures from four animals (one-way ANOVA with Tukey's post hoc test).

Development of neural connections may additionally serve as a guide to mechanisms of injury. Guidance cues are critical to pathfinding by axonal growth cones as well as synaptogenesis.^56^ Some of the signaling pathways activated by these factors are well known. Somewhat surprisingly many of these factors have been shown to increase after retinal injury and disease. For instance, semaphorin 3A (Sema 3A) increases in the retina after retinal detachment,^57^^,^^58^ optic nerve axotomy,^59^ diabetic retinopathy,^60^ OIR,^61^ and glaucoma;^62^ netrin-1 is upregulated in OIR and diabetic retinopathy;^63^^–^^66^ eph/ephrin signaling is involved in OIR and diabetic retinopathy^67^^–^^69^ and increases in glaucoma.^70^^–^^73^ In contrast, ROBO1, a receptor for the repulsive guidance cue slit, and normally present in photoreceptor terminals, decreases in disease.^22^ These changes in guidance factors have been observed in both animal models and patients. Additionally, dramatic upregulation of genes for canonical pathways of axon guidance, including for ephrin and semaphorin, is reported in a CNGA3/CNGB1 double mutant mouse that displays extensive horizontal and bipolar cell sprouting. Since guidance cues can promote both axonal and dendritic growth,^74^^,^^75^ retinal cell sprouting by secondary neurons may be influenced by these factors. In cultures of adult amphibian rod and cone photoreceptors, we found that guidance factors modulate synaptic plasticity. Sema 3A reduced rod neuritic sprouting^58^ whereas netrin-1 promoted presynaptic varicosity formation in rod but not cone cells (Fig. 2).

### Signaling Pathways

**Figure 3.**
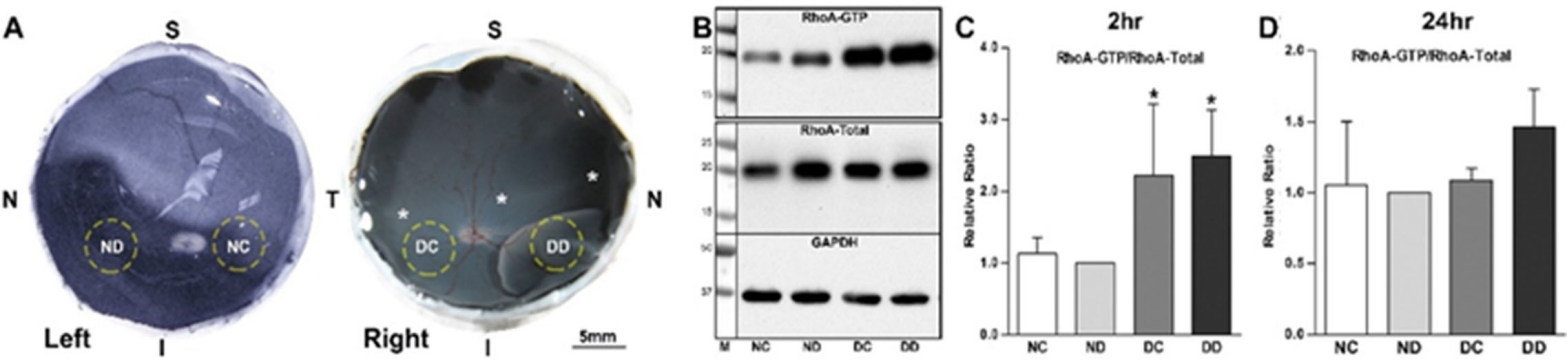
RhoA activation in porcine retina in vivo*.* **A.** Samples were taken from the detached (DD) and attached retina (DC) in the operated right eye and from the same areas in the normal (unoperated) left eye (ND, NC). **B, C**. Two hours after detachment, active RhoA (RhoA-GTP obtained with a pull-down assay) increases in DD and DC (*P < 0.05, *n* = 16 retinal samples, four pigs). **D.** RhoA activation remains above control, but lower than at two hours, in the detached area after 24 hours (*P* = 0.07, *n* = four pigs). Although activation of RhoA protein increased, total RhoA protein did not change (normalized with GAPDH). S, superior, I, inferior, N, nasal, T, temporal. *Location of cone rich area centralis. Data expressed as mean + SD; normal eye, ND, normalized to 1, one-way ANOVA. Panels A–C modified from Wang et al. 2016.^6^

**Figure 4.**
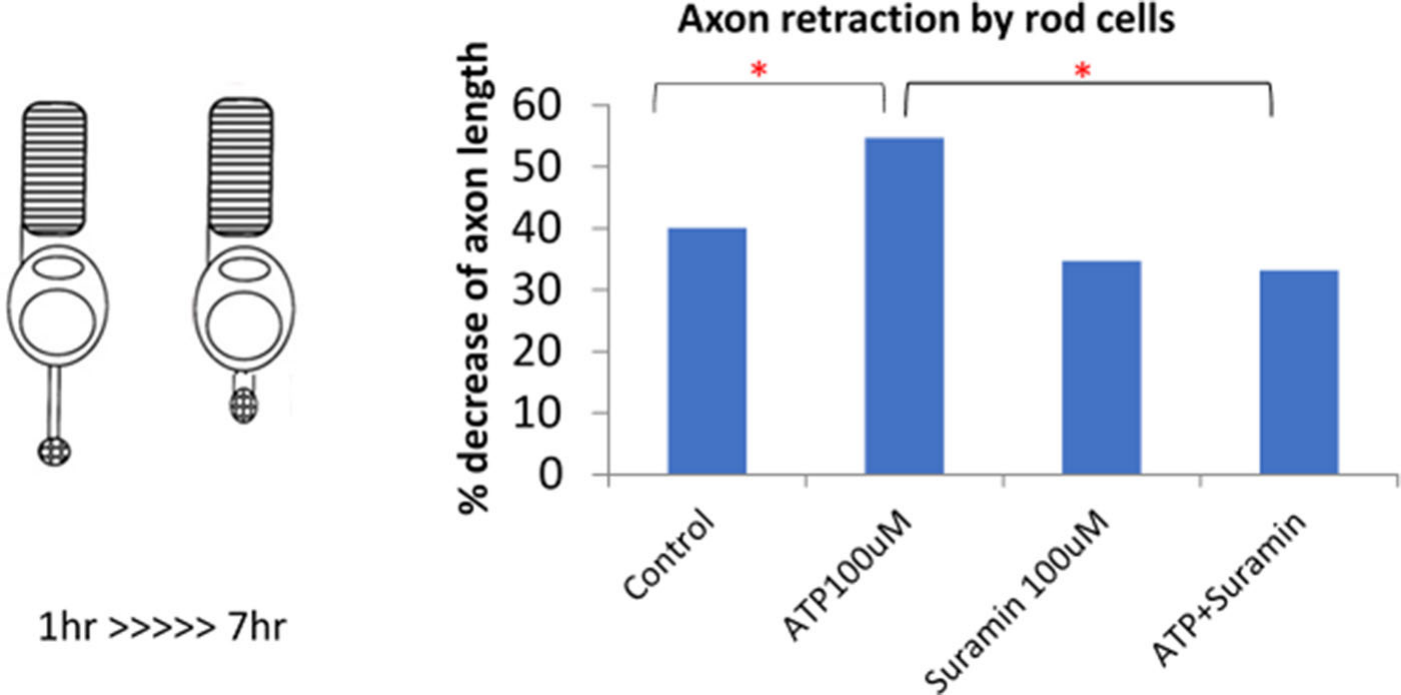
ATP promotes axon retraction in rod cells. *Left*, isolated rod cell in culture showing axon retraction over a six-hour period. *Right*, ATP increases the amount of retraction; suramin, a purinergic receptor antagonist, reduces retraction. *P < 0.05, *n* = 100 cells per condition, from five animals (one-way ANOVA with Tukey's post hoc test).

The chemorepulsive factor Sema 3A works through receptors that activate RhoA. We reported that not only did Sema 3A and its receptor neuropilin-1, present on most retinal neurons, increase after injury,^58^ so did activated RhoA, spiking after detachment but frequently remaining at above normal levels for at least 24 hours (Fig. 3). The cause for RhoA activation after retinal injury could relate to the presence of semaphorin, but additional triggers, such as mechanotransduction at the membrane that activates RhoA-associated guanine nucleotide exchange factors (GEFs)^76^ and/or injury-induced secretion of ATP, seen after mechanical stimulation and detachment in retina,^77^^,^^78^ that increases Rho kinase (ROCK) activity by binding to purinergic receptors,^79^ may also be involved. In culture, isolated rod cells retract their axonal fiber more quickly with added ATP whereas axon retraction is slowed by suramin, a purinergic antagonist (Fig. 4). Mechanotransduction and ATP secretion, which respond to injury rapidly, may be especially significant at the early times after detachment.

**Figure 5.**
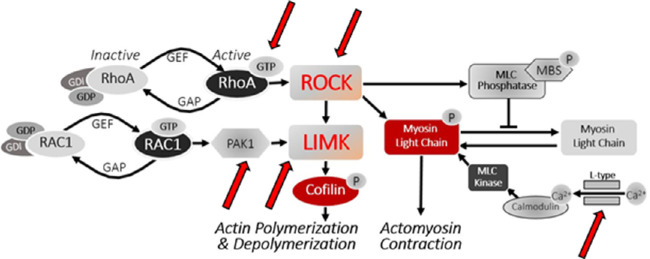
Pathway that contributes to rod synaptic disjunction after detachment. *Red arrows* point to targets of blockers tested: CT-04 against RhoA; Y27632, fasudil and AR13503 against Rho kinase (ROCK); IPA-3 against p21-activated kinase (PAK); BMS-5 against LIM kinase (LIMK); nicardipine against L-type calcium channel. All blockers reduced rod spherule retraction. Data from Nachman-Clewner et al. 1999; Zhang & Townes-Anderson 2002; Fontainhas & Townes-Anderson 2008, 2011; Wang & Townes-Anderson 2015; Wang et al. 2016; Townes-Anderson et al. 2017; Wang et al. 2019; and Halasz et al. 2021.^6^^–^^8^^,^^40^^,^^47^^,^^48^^,^^80^^–^^82^

We have reported experiments in which components of the RhoA-Rho kinase (ROCK)-LIM kinase (LIMK) pathway are blocked. In our injury models, both in vitro and in vivo,^6^^–^^8^^,^^40^^,^^49^^,^^80^^–^^82^ anything that reduced the activity of RhoA or its downstream targets reduced rod synaptic disjunction (Fig. 5). The effects of inhibitors are directly on the photoreceptor themselves, as their terminals contain RhoA and LIMK,^80^^,^^82^ although we do not rule out additional effects on other neurons, epithelial cells, and vascular endothelium. For cone cells we know that ROCK inhibition can also modify synaptic structure. RhoA is present in the pedicles of adult cone cells.^80^ In cultures of isolated salamander cones, ROCK inhibition increased neuritic growth and the development of synaptic varicosities. In our in vivo pig model, where cone neuritic growth is not seen, preliminary data indicate that ROCK inhibition prevents the reduction in size of cone synaptic ribbons that occurs in response to a 2-hour retinal detachment (unpublished data, 2021).

Signaling pathways in activity-dependent synaptic plasticity and neural development thus provide a broad canvas for experimentation on ways to preserve synaptic structure at the first synapse. However, an additional consideration could provide more focus in the search for therapeutics. Some elements in these pathways appear almost uniquely after injury. Activated RhoA, for instance, is at very low levels in the retina under normal conditions.^6^ Sema 3A is absent in the normal retina.^58^ The advantage of targets such as these is that drugs or antibodies blocking their activity are less likely to disrupt normal synaptic function. It can be likened to a conditional gene knockout, a more precise therapeutic tool. Our use of a ROCK inhibitor in retinal detachment seems to be such a focused therapy. However, discovering the timing of the upregulation of these transitory injury-induced targets will be a challenge.

## Conclusions

Determining the role of retinal synapses in visual recovery or the lack thereof clearly deserves more attention. Although advances in our understanding may depend in part on the development of new techniques to assess the structure and function of ribbon synapses in disease and injury, much can be learned by application of current high-resolution microscopy and electrophysiology. In terms of treatment, we know that the visual system can tolerate some loss of synaptic connections, perhaps, in part, because of built-in redundancy: 40% or more of cone cells can die, and a patient can retain normal visual acuity and foveal sensitivity.^83^^,^^84^ This fact may be advantageous by providing time to introduce compounds, such as ROCK inhibitors, to preserve the carefully choreographed synaptic circuitry that remains. Moreover, preservation of the outer retinal synaptic circuitry may also benefit the inner retina, which is known to undergo extensive remodeling after injury and during disease.^5^^,^^85^ As part of the central nervous system, synaptic preservation in the retina is especially critical as regeneration of appropriate connections is poor.
